# External Evaluation of Vancomycin Population Pharmacokinetic Models at Two Clinical Centers

**DOI:** 10.3389/fphar.2021.623907

**Published:** 2021-03-15

**Authors:** Yi-Xi Liu, Haini Wen, Wan-Jie Niu, Jing-Jing Li, Zhi-Ling Li, Zheng Jiao

**Affiliations:** ^1^ Department of Pharmacy, Shanghai Chest Hospital, Shanghai Jiao Tong University, Shanghai, China; ^2^ Department of Pharmacy, the First Affiliated Hospital, Zhejiang University School of Medicine, Hangzhou, China; ^3^ Department of Pharmacy, Huashan Hospital, Fudan University, Shanghai, China; ^4^ Department of Pharmacy, Suzhou Hospital Affiliated to Nanjing Medical University, Suzhou, China; ^5^ Department of Pharmacy, Shanghai Children’s Hospital, Shanghai Jiao Tong University, Shanghai, China

**Keywords:** vancomycin, population pharmacokinetics, neonates, external evaluation, individualized drug administration

## Abstract

**Background:** Numerous vancomycin population pharmacokinetic models in neonates have been published; however, their predictive performances remain unknown. This study aims to evaluate their external predictability and explore the factors that might affect model performance.

**Methods:** Published population pharmacokinetic models in neonates were identified from the literature and evaluated using datasets from two clinical centers, including 171 neonates with a total of 319 measurements of vancomycin levels. Predictive performance was assessed by prediction- and simulation-based diagnostics and Bayesian forecasting. Furthermore, the effect of model structure and a number of identified covariates was also investigated.

**Results:** Eighteen published pharmacokinetic models of vancomycin were identified after a systematic literature search. Using prediction-based diagnostics, no model had a median prediction error of ≤ ± 15%, a median absolute prediction error of ≤30%, and a percentage of prediction error that fell within ±30% of >50%. A simulation-based visual predictive check of most models showed there were large deviations between observations and simulations. After Bayesian forecasting with one or two prior observations, the predicted performance improved significantly. Weight, age, and serum creatinine were identified as the most important covariates. Moreover, employing a maturation model based on weight and age as well as nonlinear model to incorporate serum creatinine level significantly improved predictive performance.

**Conclusion:** The predictability of the pharmacokinetic models for vancomycin is closely related to the approach used for modeling covariates. Bayesian forecasting can significantly improve the predictive performance of models.

## Introduction

Vancomycin is a glycopeptide antibiotic used as the gold standard treatment for serious infections in adults caused by Gram-positive bacteria, especially methicillin-resistant *Staphylococcus aureus* (Pacifici and Allegaert, 2012). Vancomycin is also effective in infants with serious Gram-positive infections. However, the therapy window of vancomycin is narrow, and differences in neonatal development and pathophysiology result in high inter-individual variability in vancomycin pharmacokinetics (Stockmann et al., 2015). Although excessive exposure to vancomycin can lead to side effects including ototoxicity and nephrotoxicity (An et al., 2011), under-dosing is often associated with treatment failure and patient mortality (Rybak et al., 2020). Therefore, despite the challenges, it is imperative to optimize vancomycin regimens in neonates.

Therapeutic drug monitoring is an applicable approach for the pharmaceutical care of vancomycin. According to the American Society of Health-System Pharmacists consensus (2020), the administration target for vancomycin is an area under the concentration-time curve (AUC)/minimum inhibitory concentration of ≥400 h in neonates and infants (Rybak et al., 2020). Although obtaining a sufficiently large number of samples to estimate the AUC is difficult in clinical practice, especially for neonates, a population pharmacokinetic analysis could provide sufficient pharmacokinetic parameters to estimate the AUC through sparse sampling. It is possible to model vancomycin dosing in neonates through reliable individual pharmacokinetic characteristics using Bayesian approaches.

Choosing the appropriate population PK model to estimate the initial and maintenance dosage for vancomycin is essential in clinical practice; however, the performance of most of the published pharmacokinetic models is still unknown. Zhao et al. (2013a) conducted an external evaluation of six models in neonates and found that the analytical method used for serum creatinine (SCR) is a crucial factor in explaining the variability of predictions among different studies. However, more than ten population PK studies have been conducted since then, using several new modeling strategies. Therefore, it is still worth evaluating all the published population pharmacokinetic models for vancomycin in neonates.

Our research aimed to systematically evaluate the published population pharmacokinetic models of vancomycin in neonates, using data from independent cohorts collected from two clinical centers. Moreover, factors that may influence model predictability were also investigated, such as structural model selection and covariate screening approaches, to provide informed guidance for future studies.

## Materials and Methods

### Review of Published popPK Studies

The PubMed, Scopus, and Web of Science databases were systematically searched for population pharmacokinetic analyses of vancomycin published up to October 2020. The key words “vancomycin,” “pharmacokinetic” or “pharmacokinetics” or “model” or “nonlinear mixed effect model” were used in the search strategy. The publications were included if 1) the study was a population pharmacokinetic analysis of vancomycin in neonates and 2) the article was written in English.

The publications were excluded if 1) the model was not created using a nonlinear mixed-effects modeling approach, or 2) the model could not be recreated using the published information, or 3) the modeling populations overlapped or the articles were duplicated.

### External Evaluation Cohort

#### Patients

Datasets were derived from published population PK studies conducted in neonates who received vancomycin at Shanghai Children’s Hospital between January 2013 and December 2016 (Li et al., 2018), and Suzhou Hospital Affiliated to Nanjing Medical University between September 2011 and March 2016 (Li et al., 2017). Patients included in these two studies were preterm neonates with a postmenstrual age (PMA) of ≤48 weeks and term neonates with a postnatal age (PNA) of ≤28 days. All patients were treated with vancomycin for at least 3 days, and at least one vancomycin level was determined based on routine therapeutic drug monitoring. Patients with extracorporeal membrane oxygenation or who were on continuous renal replacement therapy were excluded from this study.

The following information was collected in each study: gestational age (GA), PMA, PNA, current weight (WT), birth weight, dosing records, measurements of vancomycin levels, and SCR level.

The doses of vancomycin ranged from 10 to 15 mg/kg, administered every 8 h or every 12 h with a 1 h or 2 h infusion duration. Peak samples were collected 1 h after completion of drug infusion, and trough samples were collected half an hour before vancomycin administration in each neonate. Trough and peak levels were determined after at least four repeated doses.

#### Bioassay

Vancomycin levels were determined using a fluorescence polarization immunoassay with an Architect i2000SR (Abbott Laboratories, Chicago, IL, UNITED STATES). The limit of detection was 1 mg/L, and the calibration range was 3–50 mg/L. The intra-day and inter-day coefficients of variation were <20%.

SCR assays were performed at the Shanghai Children’s Hospital using the enzymatic method and were analyzed with a 7,180 automatic analyzer (Hitachi High-Tech Science Systems Corporation, Tokyo, Japan). The calibration range was from 3 to 100 mg/L. SCR assays were performed at the Suzhou Hospital Affiliated to Nanjing Medical University using the enzymatic method and were analyzed with a 7,600 Automatic Analyzer (Hitachi High-Tech Science Systems Corporation, Tokyo, Japan). The calibration range was 0.08–100 mg/L.

Creatinine clearance was calculated using the Schwartz formula as in Eq. 1 (Schwartz et al., 1984):

$$CLcr(mL/min/1.73\times m^{2})=\frac{k\times HT}{SCR},$$ (1)

where CLcr represents creatinine clearance, HT (cm) represents height, SCR (umol/L) represents serum creatinine, k is 0.45 for term neonates, and 0.33 for preterm neonates.

SCR was standardized to the enzymatic method (SCR*) if the Jeff method (SCR^†^) was employed in the external model by Eq. 2 (Srivastava et al., 2009)

$$\text{SCR}\times \left(\text{μmol}/\text{L}\right)=1\text{.}050\times 88\text{.}41\times {\text{SCR}}^{\text{†}}\times \left(\text{mg}/\text{dL}\right)-\text{0}\text{.}122\text{.}$$ (2)

If the method was not clarified in the report, the enzymatic method was used.

### External Evaluation

Data were analyzed using a nonlinear mixed-effects modeling program (NONMEM®, Version 7.4; Icon Inc., PA, UNITED STATES) compiled with gFortran (Version 4.9.2; http://www.gfortran.org). Statistical analysis and graphing were performed using R (Version 3.6.1; http://www.r-project.org) and the xpose package.

The reported population pharmacokinetic model was reconstructed based on information extracted from the original articles. The NONMEM code for each model was determined by a double check. The predictabilities of all candidate models were externally evaluated by prediction- and simulation-based diagnosis and Bayesian forecasting (Zhao et al., 2016; Mao et al., 2018; Cai et al., 2020).

#### Prediction-Based Diagnostics

The predicted population concentrations (PRED) were estimated and compared with the corresponding observations (OBS) by estimating the relative prediction error (PE%) using Eq. 3:

$$\text{ PE}\left(\%\right)=\frac{\text{ PRED}-\text{OBS}}{\text{OBS}}\times 100\%.$$ (3)

The median prediction error (MDPE) was used to evaluate predictive accuracy, whereas the median absolute prediction error (MAPE) was used to evaluate predictive precision. F_20_ and F_30_ were also calculated as combination indexes of both accuracy and precision, and indicate the percentage of PE that fell within the ±20% and ±30% ranges, respectively. When the standards of MDPE ≤ ± 15%, MAPE ≤30%, F_20_ > 35%, and F_30_ > 50% were reached, the model could be determined as satisfactory and clinically acceptable.

#### Simulation-Based Diagnostics

A prediction- and variability-corrected VPC (pvcVPC) (Bergstrand et al., 2011) was conducted for simulation-based diagnostics. The pvcVPC takes into account typical population predictions and typical population variabilities compared with the traditional VPC, accounting for the different expected variabilities within individuals. The pvcVPC was conducted with 1,000 simulated datasets generated using the models to be evaluated. The pvcVPC was performed using the Perl speak NONMEM toolkit (PsN, version 4.7.0).

Maximum a posteriori Bayesian (MAPB) forecasting was conducted to assess the influence of prior observations on model predictability. Patients with ≥1, 2, three observations were included in the analysis for Bayesian forecasting using zero, one and two previous observations, respectively. For a patient, the individual prediction (IPRED) of the third observation was predicted using the first and second observations, the second observation was predicted using the first observation, and then compared with the corresponding observations. The relative differences denoted by the individual prediction error (IPE%) were calculated using Eq. 4 below:

$${\text{IPE}}_{i}\left(\%\right)=\frac{{\text{IPRED}}_{i}-{\text{OBS}}_{i}}{{\text{OBS}}_{i}}\times 100\left(i=1,2,3\right).$$ (4)

To evaluate the predictability of the candidate models when prior information is increased, the standards of an IPE% (MDIPE) ≤ ± 15%, an IPE% (MAIPE) ≤ 30%, an IF_20_ > 35%, and an IF_30_ > 50% were used for MAPB forecasting.

#### The Impact of Modeling Approaches

Different modeling strategies were used in previous studies, which may affect the predictive performance of the model. To explore the impact of these different modeling strategies, we evaluated the predictability of various structural models and covariate models employed in previous studies. The assessment methods include the aforementioned prediction-based diagnostics and Bayesian forecasting methods.

## Results

### Review of Published popPK Analysis on Vancomycin

After a systematic literature search, 18 neonatal vancomycin models (Seay et al., 1994; Grimsley and Thomson, 1999; Capparelli et al., 2001; Kimura et al., 2004; Mulla and Pooboni, 2005; Marqués Minñana et al., 2010; Mehrotra et al., 2012; Zhao et al., 2013b; Frymoyer et al., 2014; Li et al., 2017; Sheng et al., 2017; Song et al., 2017; Chen et al., 2018; Li et al., 2018; Moffett et al., 2018; Colin et al., 2019; Germovsek et al., 2019; Moffett et al., 2019) were included in this study. The literature search procedure is shown in Supplementary Figure S1. Of the enrolled studies, six were from UNITED STATES, three from the UNITED KINGDOM, five from China, one each from Japan and Spain, and one study enrolled patients from France, Greece, France, and Malaysia. Only 10 models described the analytical method used for the determination of SCR.

Most studies employed sparse sampling strategies. Six models were established with a two-compartment model (2CMT), whereas 12 models were established with a one-compartment (1CMT) model.

Weight, age, and renal function were the most important covariates for clearance identified in the previous studies. Maturation models were employed in 11 studies and could be described by Eq. 5 (Holford et al., 2013):

$$\text{CL}={\text{CL}}_{\text{std}}\times {\text{F}}_{\text{size}}\times {\text{F}}_{\text{mat}},$$ (5)

where CLstd represents the baseline clearance, Fsize refers to the body size factor, and Fmat refers to the maturation factor.

Weight (current weight and birth weight) and age (postmenstrual age, PMA; postnatal age, PNA, and GA) were regarded as the main factors for body size and maturation, respectively. The current weight was used in most of the reported studies. PMA was chosen over GA and PNA as it presented the most parsimonious way to account for both antenatal and postnatal maturation, which can be incorporated as a sigmoid Emax model and asymptotic exponential model, as shown in Eq. 6 and Eq. 7 (Salman et al., 2019). Among the included studies, seven out of 18 models applied the sigmoid Emax model:

$$\text{Fmat}=\frac{1}{1+{\left(\frac{\text{PMA}}{{\text{TM}}_{50}}\right)}^{\text{Hill}}},$$ (6)

where TM_50_ is the value of PMA when clearance maturation reaches 50% of adults; Hill is the slope parameter for the sigmoid E_max_ maturation model.

$$\text{Fmat}={\text{e}}^{\theta \exp\times \left(PMA-medianPMA\;\right)}.$$ (7)

Renal function was often presented by SCR in reported studies and was included in nonlinear manner.

The characteristics of each study are summarized in Table 1, and the information on population pharmacokinetic models were shown in Table 2.

**TABLE 1:** Summary characteristics of published population pharmacokinetic studies of vancomycin in neonates.

Author	Country	Patients/Samples	SCR (μmol/L) (median, min- max)	WT (kg) (median, min- max)	PMA (weeks) (median, min- max)	PNA(days) (median, min- max)	GA (weeks) (median, min- max)	Serum creatinine measurement
Seay_et al., 1994	UNITED STATES	192/520	NA	1.48 (0.39–4.35)	NA	14.5 (1–73)	29.6 (22–42)	NA
Grimsley and Thomson, 1999	UNITED KINGDOM	59/347	49.0 (18.0–172)	1.52 (0.57–4.23)	NA	19 (2–76)	29 (25–41)	Jaffe method
Capparelli et al., 2001	UNITED STATES	374/1,103	66.9 (NA)	2.82 (NA)	NA	70 (NA)	33.5 (NA)	Jaffe method
Kimura et al., 2004	Japan	19/88	17.7–79.6	NA (0.710–5.20)	NA	NA (3.00–71.0)	NA (24.1–41.3)	Enzymatic method
Mulla and Pooboni, 2005	UNITED KINGDOM	15/NA	79.6 (39.0–180)	3.50 (2.50–4.50)	NA	8.20 (0–28.0)	40.4 (34.3–42.0)	NA
Marqués Minñana et al., 2010	Spain	70/NA	NA	1.70 (0.70–3.70)	34.6 (25.1–48.1)	16.9 (4.00–63.0)	32.2 (24.0–42.0)	NA
Mehrotra et al., 2012	UNITED STATES	134/267	53.1 (17.7–221)	2.50 (0.60–5.30)	36.5 (24.6–44)	26.8 (1–121)	32.7 (23–41)	NA
Zhao et al., 2013a	France	116/207	48.0 (5.00–228)	1.70 (0.46–5.68)	33.8 (24.4–49.4)	26 (1–120)	NA	NA
Frymoyer et al., 2014	UNITED STATES	249/1702	NA (8.8–239)	2.90 (0.500–6.30)	39 (24–54)	19 (0–173)	34 (22–42)	Jaffe method
Li et al. ., 2017	China	80/165	28.3 (5.85–61.6)	2.74 (1.4–5.6)	40.0 (29–47.1)	24 (4–126)	34 (25.7–41.1)	Enzymatic method
Sheng et al., 2017	China	61/72	32.3 (10.4–109)	3.15 (0.95–16.0)	NA	29 (1–354)	NA	Jaffe method
Song et al., 2017	China	102/316	28.6 (12–151)	3.95 (1.25–7.62)	NA	NA	37 (28–41)	NA
Chen et al., 2018	China	213/330	24.8 (9.72–63.7)	2.73 (0.88–5.1)	39.8 (28–47.9)	26 (6–59)	24.8 (9.72–63.7)	NA
Li et al., 2018	China	80/165	32.2 (13.1–54.2)	1.9 (0.81–4.71)	35.02 (28.3–44.0)	17 (4–50)	32.6 (25.7–41.3)	Enzymatic method
Moffett et al., 2018	UNITED STATES	93/NA	49.5 (28.2–89.3)	7.6 (3.7–21.9)	73.2 (41.1–391.2)	233 (25.6–2,446)	49.5 (28.2–89.3)	NA
Colin et al., 2019	Belgium	247/NA	64.5 (34.5–187)	1.20 (0.42–2.63)	31.3 (24–37)	11 (1–27)	NA	NA
	Greece	130/NA	50.4 (23–180)	1.07 (0.51–4.41)	31.3 (26.6–43.8)	13 (3–27)	NA	NA
	France	67/NA	53 (17.7–274.8)	1.06 (0.68–4.45)	31.3 (27.1–45.9)	13 (4–95)	NA	NA
	Malaysia	116/NA	77.8 (29.2–143)	0.90 (0.50–2.00)	28.7 (23.5–33.9)	5 (1–39)	NA	NA
Germovsek et al., 2019	UNITED KINGDOM	54/102	31.0 (18–98)	NA	29 (23.7–41.9)	30 (1–156)	NA	Jaffe method
Moffett et al., 2019	UNITED STATES	261/NA	28.3 (22.1–36.2)	4.8 (3.4–7.4)	54.6 (42.6–76.9)	27 (26–281)	38.7 (37.1–40)	NA

GA, gestational weeks (weeks); PMA, postmenstrual age (weeks); PNA, postnatal age (days); Scr, serum of creatinine (μmol/L); WT, weight (kg); NA, not available.

**TABLE 2:** Summary models’ information of published population pharmacokinetic studies of vancomycin in neonates.

Author	Structural model	Parameter and formula		BSV% (IOV%)	Residual error
Seay et al.,1994	2CMT	CL	0.059 × WT × 0.46 (if co-therapy with dopamine) × 0.643 (If GA≤32)	40.6%	3.3 mg/L
		V_C_	0.44 × WT	16.8%	
		V_SS_	0.769 × WT	54.1%	
		Q	0.0313 × WT	/	
Grimsley et al., 1999	1CMT	CL	3.56 × WT/ [(SCR+0.12)/1.05]	22%	4.53 mg/L
		V	0.669 × WT	18%	
Capparelli et al., 2001	2CMT	CL	0.006 + WT × [0.028/SCR + 0.000127 × PNA + 0.0123 (If GA>28)]	32%	14%, 3.4 mg/L
		Vss	0.793 × WT +0.01	16%	
		Vc	0.0666 × (0.793 × WT +0.01)	/	
		Q	0.0334 × WT	/	
Kimura et al., 2004	1CMT	CL	0.025 × WT× (88.41/ SCR) × 1.292 (If PCA ≥34 weeks)	22.9%	3.22 mg/L
		V	0.66 × WT	20.8%	
Mulla et al., 2005	2CMT	CL	WT / [(SCR+0.12)/1.05] × 4.3(If PNA >1000 days) × (2.4 + 0.0018 × PNA) (If PNA <1000 days)	25%	12.1%, 2.1 mg/L
		Q	0.09 × WT	91%	
		V_c_	WT× 0.45 (If PNA <4000 days) × 0.37 (If PNA >4000 days)	25%	
		V_T_	0.25 × WT	48%	
Marqués Minñana et al., 2010	1CMT	CL	0.00192× PMA× WT× 1.65 (If co-therapy with amoxicillin–clavulanic acid)	35.6%	2.69 mg/L
		V	0.572× WT × 0.656 (If co-therapy with spironolactone)	19.3%	
Mehrotra et al., 2012	1CMT	CL	0.18 × (WT/2.5)^0.75^× (0.42/[(SCR/88.41+0.12)/1.05])^0.7^ × (PMA/37)^1.4^	25.3%	1.5 mg/L,16% (if LOQ = 0.5 mg/L); 5 mg/L(if LOQ = 5 mg/L)
		V	1.7× (WT/2.5)^1.0^	21.8%	
Zhao et al., 2013	1CMT	CL	0.0571× (WT/1.416)^0.513^× (bWT/1.01)^0.599^ × (1+0.282× PNA/17) × 1/(SCR/42)^0.525^	40.1%	20.3%, 2.28 mg/l
		V	0.791 × (WT/1.416)^0.898^	17.9%	
Frymoyer et al., 2014	1CMT	CL	0.345× (WT/2.9)^0.75^ × 1/[1+(PMA/34.8)^-4.53^] × (1/[(SCR/88.41+0.12)/1.05])^0.267^	21.6%	20.5%, 1.3 mg/L
		V	1.75 × (WT/2.9)	10.9%	
Li et al., 2017	1CMT	CL	4.6× (WT/70)^0.75^ × [PMA^5.46^/(PMA^5.46^+37.6^5.46^)]× 1.230/SCR	24.4%	36.9%
		V	61.1× (WT/70)	/	
Sheng et al., 2017	1CMT	CL	0.449× (WT/3.22)^0.0643^× (PNA/36.5)^0.289^	13.9%	0.281 mg/L
		V	4.45	/	
Song et al., 2017	2CMT	CL	0.42× (bWT /3.22)^0.888^× (PNA/29)^0.449^	46.60%	1.48 mg/L
		Vc	1.27	/	
		Vp	2.422	/	
		Q	1.161	/	
Chen et al., 2018	1CMT	CL	4.87× (WT/70)^0.75^ × [PMA^4.61^/(PMA^4.61^+34.5^4.61^)] × [(SCR/88.41+0.12)/1.05]^-0.221^	26.80%	23.9%, 0.688 mg/L
		V	40.7× (WT/70)	/	
Li et al., 2018	1CMT	CL	0.309× (WT/2.9)^1.55^ × (SCR/23.3)^-0.337^	37.3%	37.9%
		V	2.63× (WT/2.9)^1.05^	/	
Moffett et al., 2018	2CMT	CL	3.96× (WT/70)^0.75^× [0.588/(SCR/88.41+0.12)]^0.809^× [1/(1+(PMA/43)^-0.949^]	28.8%	19.40%
		Vc	25.2× (WT/70)× 0.932^(PNA/233.6)^	94.8%	
		Vp	32.4× (WT/70)× 1.27^(2.9/ALB)^	/	
		Q	5.8	/	
Germovsek et al., 2019	1CMT	CL	5.7× (WT/70)^0.632^× [PMA^3.33^/(PMA^3.33^+55.4^3.33^)]	31.6% (30%)	30%
		V	39.3× (WT/70)	31.6%	
Moffett et al.,2019	1CMT	CL	7.86× (WT/70)^0.75^× (CLCR/84)^0.9^ × [1/(1+(PMA/50)^-0.285^]	17.4%	19.90%
		V	63.6× (WT/70)	25.5%	
Colin et al., 2019	2CMT	CL	5.31 × (WT/70 )^0.75^ × 1/[1+(46.4/PMA)^2.89^] × 1/[1+(61.6/(PMA*0.019)^-2.24^] × e^[-0.649× (SCR/88.41-SCRstd)]^ × 1.292(if haematological malignancies)] × 0.755 (if heel-prick sampling) SCRstd^=^ e^[-1.228+log10 (PMA*0.019)× 0.672+6.27× e(3.11× PMA*0.019)]^	27.9%	21.5%, 1.23 mg/L
		Vc	42.9 × (WT/70) × 0.688 (if heel-prick sampling)	27.3%	
		Vp	41.7 × (WT/70)	97.9%	
		Q	3.22 × (WT/70)^0.75^× 0.403 (if heel-prick sampling)	/	

ALB, albumin(g/L); BSV%, the percentage of the value of between subject variation; bWT, birth weight(kg); CMT, compartment; CL, clearance (L/hour); CLCR, creatinine clearance(mL/min); GA, gestational weeks (weeks); IOV, inter-occasion variation; LOQ, limit of quantitation; PCA, postconceptional ages (weeks); PMA, postmenstrual age (weeks); PNA, postnatal age (days); Q, inter-compartmental clearance (L/hour); SCR, serum of creatinine (μmol/L); V, volume of distribution (L); Vc, volume of distribution of central compartment (L); Vss, volume of distribution of steady state; VP, volume of distribution of peripheral compartment (L); VT, the volume of tissue compartment; WT, weight (kg). /: not available.

### External Evaluation Cohort

The study population consisted of 171 neonates in whom there were 319 assessments of vancomycin levels. Of these, 80 neonates with 165 vancomycin levels were from Shanghai Children’s Hospital (SH), and 91 neonates with 154 vancomycin levels were from Suzhou Hospital Affiliated to Nanjing Medical University (SZ). The proportion of preterm neonates was larger in the SZ group than in the SH group. The patient demographics of both cohorts are shown in Table 3.

**TABLE 3:** Neonatal characteristics in external evaluation dataset.

Center	SH		SZ		Total dataset	
Variable	Mean ± SD	Median (range)	Mean ± SD	Median (range)	Mean ± SD	Median (range)
No. of patients (male/female)	80 (54/26)	—	91 (54/37)	—	171 (108/63)	—
No. of serum concentration measured	165	—	154	—	319	—
Weight (kg)	2.87 ± 0.89	2.74 (1.4–5.6)	2.19 ± 0.90	1.9 (0.81–4.71)	2.55 ± 2.42	2.42 (0.81–5.6)
Birth weight (g)	2,393 ± 968	2,410 (850–4,000)	2006.48 ± 871.88	1700 (740–3,700)	2,186 ± 907	1,000 (740–4,000)
Height (cm)	46.8 ± 4.72	47 (37–65)	43.26 ± 5.48	42 (26–53)	44.7 ± 5.27	45 (26–65)
Postnatal age, PNA (days)	32.3 ± 24.1	24 (4–126)	18.46 ± 10.28	17 (4–50)	24.7 ± 17.3	20 (4–126)
Gestational weeks, GA (week)	34.7 ± 4.31	34 (25.7–41.1)	33.32 ± 4.11	32.6 (25.7–41.3)	34.1 ± 4.08	33.1 (25.7–41.3)
Postmenstrual age, PMA (week)	39.4 ± 3.60	40.0 (29–47.1)	35.95 ± 3.96	35.02 (28.27–43.99)	37.6 ± 4.04	37.8 (28.27–47.1)
Serum creatinine^a^ (μmol/L)	23.2 ± 10.4	28.3 (5.85–61.6)	31.85 ± 9.70	32.20 (13.05–54.2)	26.4 ± 10.4	26.4 (13.05–61.6)
Creatinine clearance (ml/min/1.73m^2^)	52.3 ± 17.5	51.4 (21.8–92.5)	36.21 ± 14.88	32.09 (17.63–87.63)	41.2 ± 17.9	45.4 (17.63–92.5)
Blood urea nitrogen, BUN (mmol/L)	4.96 ± 3.89	4.10 (0.40–28.5)	4.01 ± 2.89	3.17 (0.46–15.87)	4.1 ± 3.0	3.49 (0.46–28.5)
First dosage (mg)	45.0 ± 16.4	42 (20–105)	32.20 ± 16.32	25 (8–85)	34.3 ± 17.1	30 (8–105)
Trough concentration (mg/L)	11.2 ± 7.92	9.15 (3.14–42.9)	12.17 ± 6.78	10.48 (3.32–32.23)	11.7 ± 7.37	9.94 (3.32–42.9)
Peak concentration (mg/L)	22.3 ± 11.0	20.3 (4.09–51.9)	—	—	22.3 ± 11.0	20.3 (4.09–51.9)
Albumin, ALB (g/L)	32.4 ± 5.49	32.0 (21.6–46.8)	30.40 ± 4.85	30.49 (13.39–44.09)	31.2 ± 4.80	31.4 (13.39–46.8)

^a^ Schwartz Equation.

### External Predictive Evaluations

#### Prediction-Based Diagnostics

**FIGURE 1:**
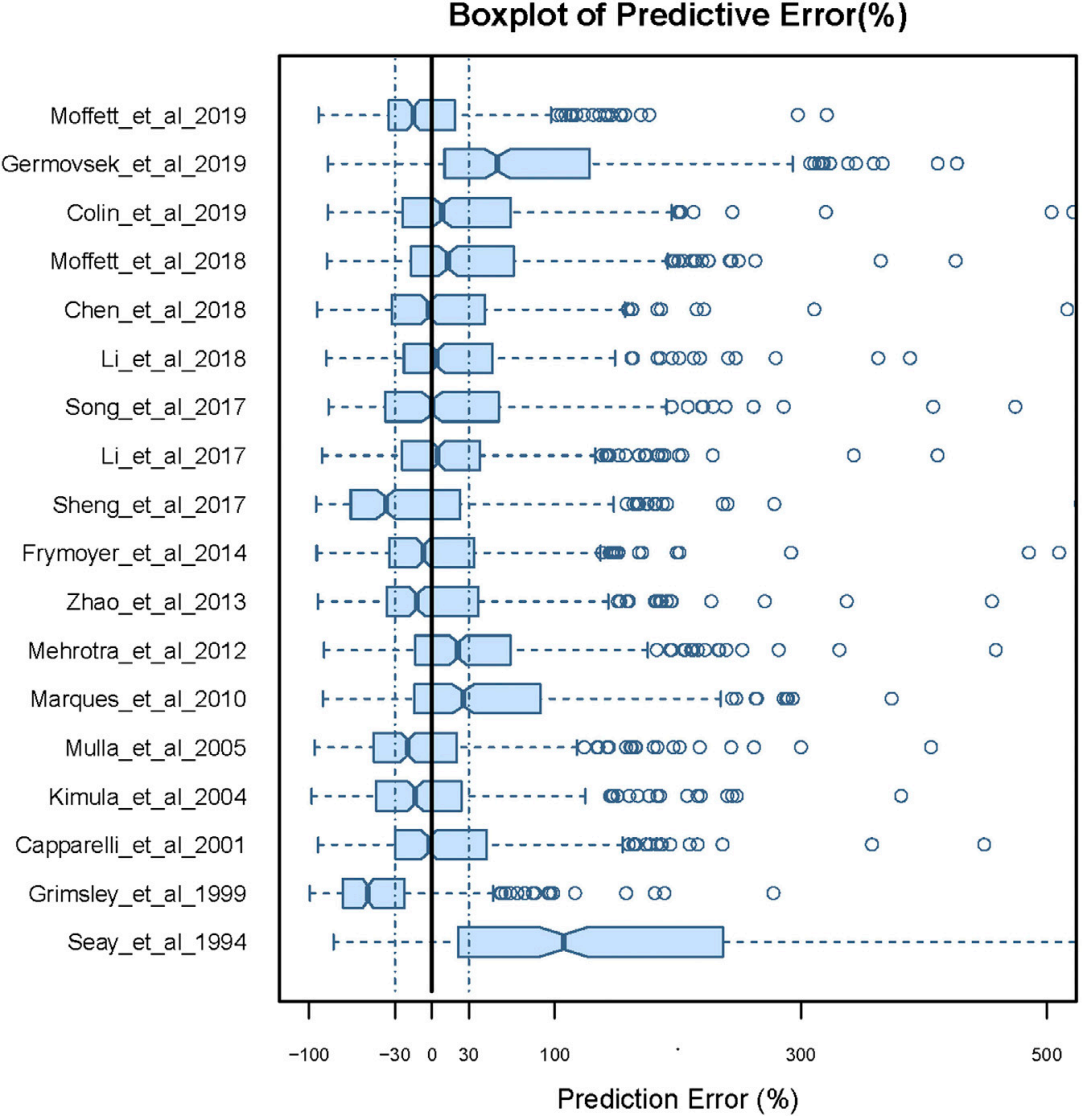
Box plots of the prediction error (PE%) for published population pharmacokinetic models in external data. Black solid lines and blue dotted lines are reference lines indicating PE% of 0% and ±30%, respectively.

There were large differences in predictability of the different models. As can be seen from the results of the prediction-based diagnostics shown in Figure 1 and Supplementary Table S1, no model satisfied the standards of MDPE ≤ ± 15%, MAPE ≤30%, F_20_ > 35%, and F_30_ > 50%. Nine models showed good predictive accuracy, with an MDPE of less than ±15%. However, MAPE was more than 30% in all models, indicating a poor predictive precision for all models. Of note, the model reported by Moffett et al. (2019) (Moffett et al., 2019) reached the criteria of F_20_ > 30% and F_30_ > 45%, showing better accuracy and precision of predictability than other models. The boxplot for PE% for each model is shown in Figure 1.

#### Simulation-Based Diagnostics

In the case of simulation-based diagnosis, the pvcVPC showed significant differences between observations and model simulations in all reported studies (Supplementary Figure S2). A clear tendency of either over- or under-prediction was observed for all models.

#### Bayesian Forecasting

**FIGURE 2:**
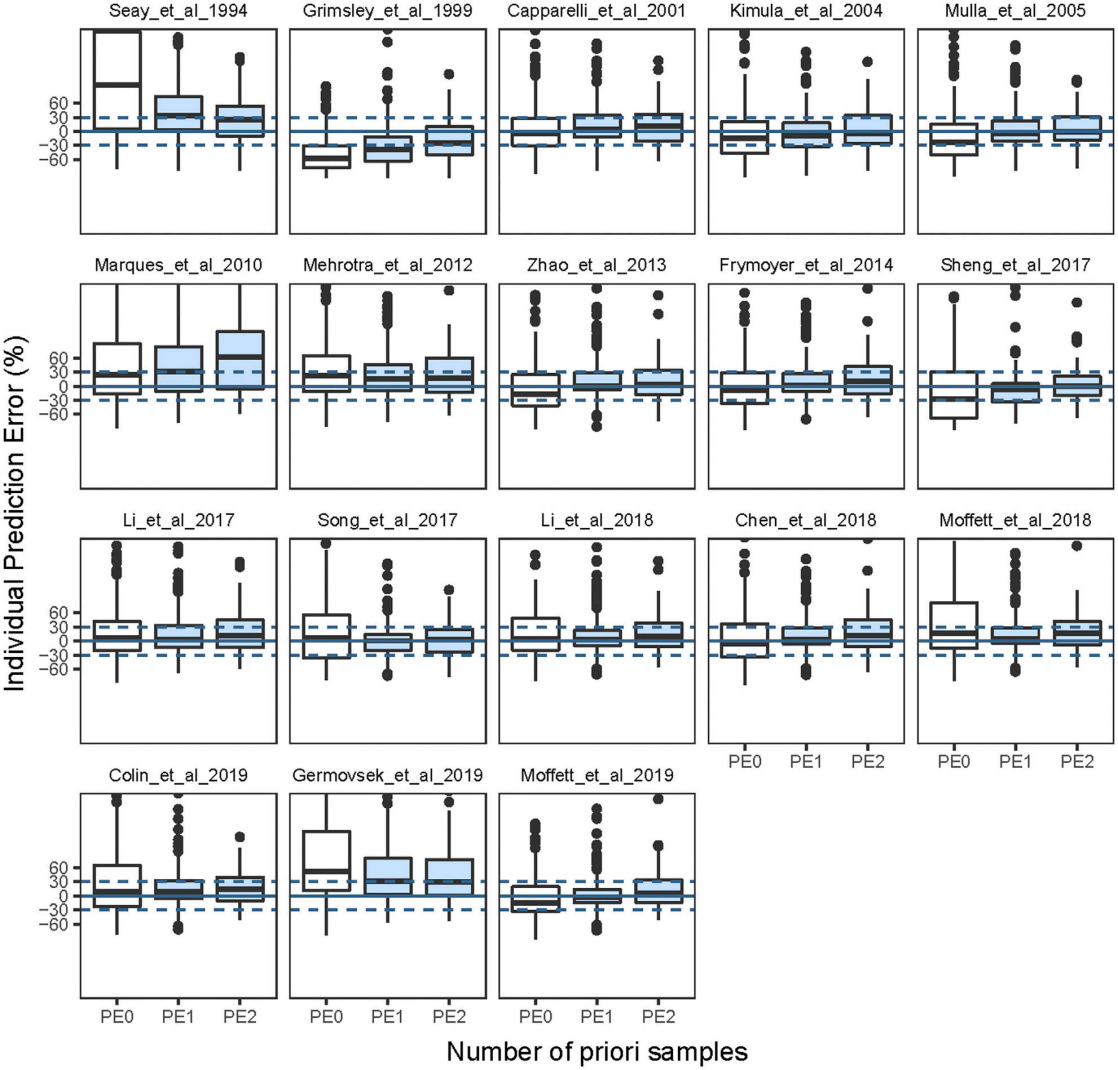
Box plots of individual prediction error (IPE%) in external data with Bayesian forecasting for published population pharmacokinetic models in different scenarios (0 represents predictions without *prior* information and one to two represents with *prior* one to two observations, respectively).

In total, 171 neonates and 171 observations, 112 neonates and 224 observations, 24 neonates and 72 observations with zero, one, two previous samples, respectively, were included in the Bayesian forecasting. After Bayesian forecasting with one or two prior observations for all models, the mean values of MDPE, MAPE, F_20_, and F_30_ compared favorably with the prediction-based diagnostics, indicating that the predictive performances had improved, as shown in Supplementary Table S2. Furthermore, 12 of the published models showed a median IPE <20%, a median absolute IPE <30%, an IF_20_ > 35%, and an IF_30_ > 50% after Bayesian forecasting with one or two prior observations. The box plots for predictability are shown in Figure 2, and the predicted indices are listed in Supplementary Table S2.

#### The Impact of Modeling Approaches

**FIGURE 3:**
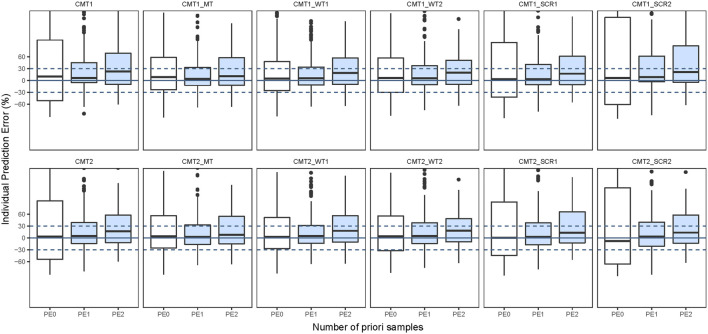
Box plots of individual prediction error (IPE%) in external data with Bayesian forecasting for one compartmental model (CMT1) and two compartmental model (CMT2) and including maturation model (MT), WT on nonlinear model (WT1) and WT on linear model (WT2), SCR on nonlinear model (SCR1) and SCR on linear model (SCR2) in external vancomycin data (0 represents predictions without *prior* information and one to two represents with *prior* one to two observations, respectively).

The structural model employed in the previous studies included the 1CMT model or 2CMT model. We evaluated the predictive performance of these two models by establishing 1CMT and 2CMT base models using the external data. Because trough concentrations could not fully describe a two-compartment model, the volume of distribution of the central compartment was fixed at 1.27 L, and the inter-compartmental clearance was fixed at 1.161 L/h in the 2CMT model, according to the study by Song et al. (Song et al., 2017). The MDPE of the 2CMT model was less than that of the 1CMT model (3.49% vs. 10.33%), indicating that the predictive accuracy was better for the 2CMT model (Figure 3 and Supplementary Table S3).

To assess the predictive performance of the various covariate models, we developed models with the identified covariates (WT, PMA, and SCR) and corresponding formulas (a maturation model, a nonlinear model, and a linear model) based on the 1CMT or 2CMT structural models. The maturation model was used by Eq. 6, nonlinear model used by Eq. 8, and linear model used by Eq. 9.

$${\text{P}}_{\text{i}}=\text{ TV}\left(\text{P}\right)\text{×}{\left(\frac{\text{COV}}{{\text{COV}}_{median}}\right)}^{\text{ θ}},$$ (8)

$${\text{P}}_{\text{i}}=\text{ TV}\left(\text{P}\right)\times \text{COV}.$$ (9)

After incorporating body size into the maturation model, the F_20_ and F_30_ were improved significantly compared to the base 1CMT model (F_20_: 27.65% vs. 14.12% and F_30_: 44.71% vs. 19.41%), or that of the 2CMT model (F_20_: 30.0% vs. 14.12% and F_30_: 43.53% vs. 19.41%). Moreover, the predictive performance of the model with WT and SCR included in a nonlinear fashion was much better than in the model where they were included in a linear fashion (Figure 3 and Supplementary Table S2). With one prior observation, the IF_20_ and IF_30_ values of the base model after Bayesian forecasting were all >35% and 50%, respectively, demonstrating an obvious improvement using Bayesian forecasting. Box plots for the predictive performance in each model are presented in Figure 3.

## Discussion

This study performed a comprehensive external evaluation of the published population pharmacokinetic models of vancomycin in neonates. Based on prediction- and simulation-based diagnostics, none of the published models had a good predictive performance according to pre-specified standards. However, after Bayesian forecasting with one or two prior measurements of vancomycin levels, the predicted performance improved significantly. This finding is consistent with external evaluation studies of other antibiotics such as rifampicin, voriconazole, and tobramycin (Cheng et al., 2020), and immunosuppressive agents (Zhao et al., 2016; Mao et al., 2018; Cai et al., 2020).

Body size is a pivotal index for the CL and V of vancomycin in neonates. Comparing different covariate modeling approaches, nonlinear models, especially the maturation model, showed much better predictability than the linear model. When the maturation model was adopted, F_20_ and F_30_ improved by 30%–50% compared with the linear model.

For drugs with narrow therapeutic windows, weight-based dosing is most commonly used for neonates because it is easy to perform. However, some studies have reported that there are adverse drug reactions related to weight-based dosage regimens for children, especially for drugs with narrow therapeutic ranges, leading to ineffective treatment outcomes and even fatalities in some cases (Koren et al., 1988; Konstan et al., 1991; Back et al., 2019).

As body weight does not fully describe organ maturity, the maturation model could better explain the physiological status of early, slower growth and subsequent faster growth in neonates (Back et al., 2019). It also allows for a quantification of the relationship between the mass/structure of organs and size (Fsize), which is known to exhibit a nonlinear pattern of growth in neonates (Holford et al., 2013) (Back et al., 2019). Moreover, published population PK analyses that included body size in the maturation models report better forecasting and better clinical use (Andrews et al., 2018; Béranger et al., 2019).

Renal function is also a very important factor affecting the pharmacokinetics of vancomycin, since vancomycin is mainly eliminated via the kidney. Creatinine clearance is used as index to describe renal function in adult patients; however, in neonates, SCR levels are a more reliable indicator of renal function, which is consistent with the findings of previous population PK studies. It has previously been shown that incorporating SCR in CL in a nonlinear fashion is better than incorporating it in a linear fashion. This finding also supports the fact that renal function matures in a nonlinear manner in neonates.

Our study has several limitations. As mentioned previously, the creatinine determination method (Jeff and enzymatic method) has been shown to have a large impact on external predictability (Zhao et al., 2013a). In this study, although we used correction equations to reduce variation between the two methods, several of the previous studies did not clearly state the method used for creatinine determination; therefore, this should be noted in future studies. Moreover, only peak and trough data were collected, and the comparison between the 1CMT and 2CMT models was not fully assessed and so may require further investigation.

## Conclusion

Based on our study, the published models performed poorly in prediction-based and simulation-based diagnostics. The maturation model based on weight, age, and nonlinear incorporation of SCR had better predictability than other modeling approaches. Moreover, the Bayesian method significantly improved the predictive performance of the published models, and could thus play an important role in vancomycin dosing recommendations and guiding clinical practice.

## Data Availability

The raw data supporting the conclusion of this article will be made available by the authors, without undue reservation.
