# Supplementary figures and images for: External Evaluation of Vancomycin Population Pharmacokinetic Models at Two Clinical Centers
Source: Front Pharmacol. 2021 Mar 15;12:623907. doi: 10.3389/fphar.2021.623907 (PMC8058705; doi:10.3389/fphar.2021.623907)

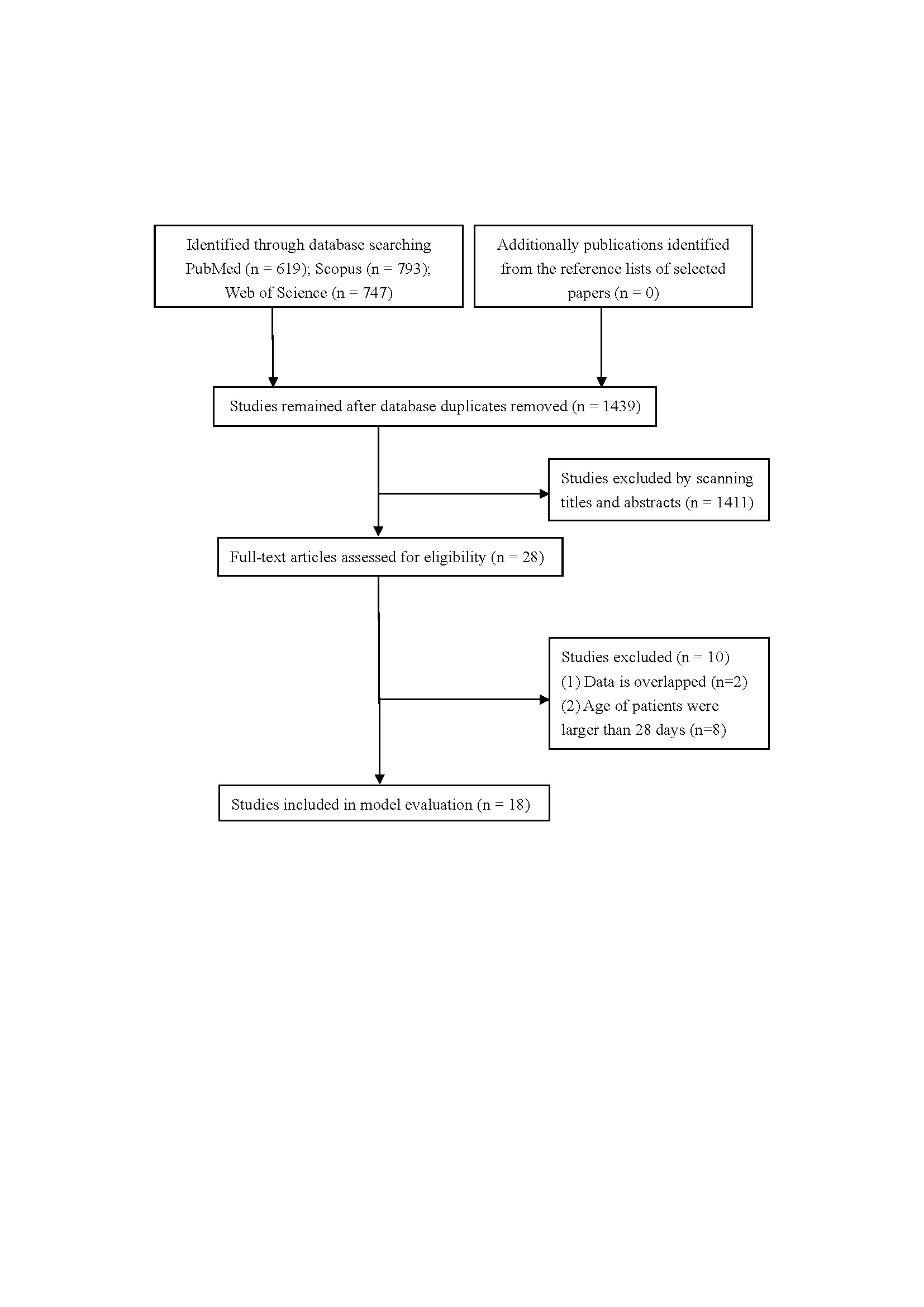

Supplement: Supplementary file 1 [file image1.jpeg]

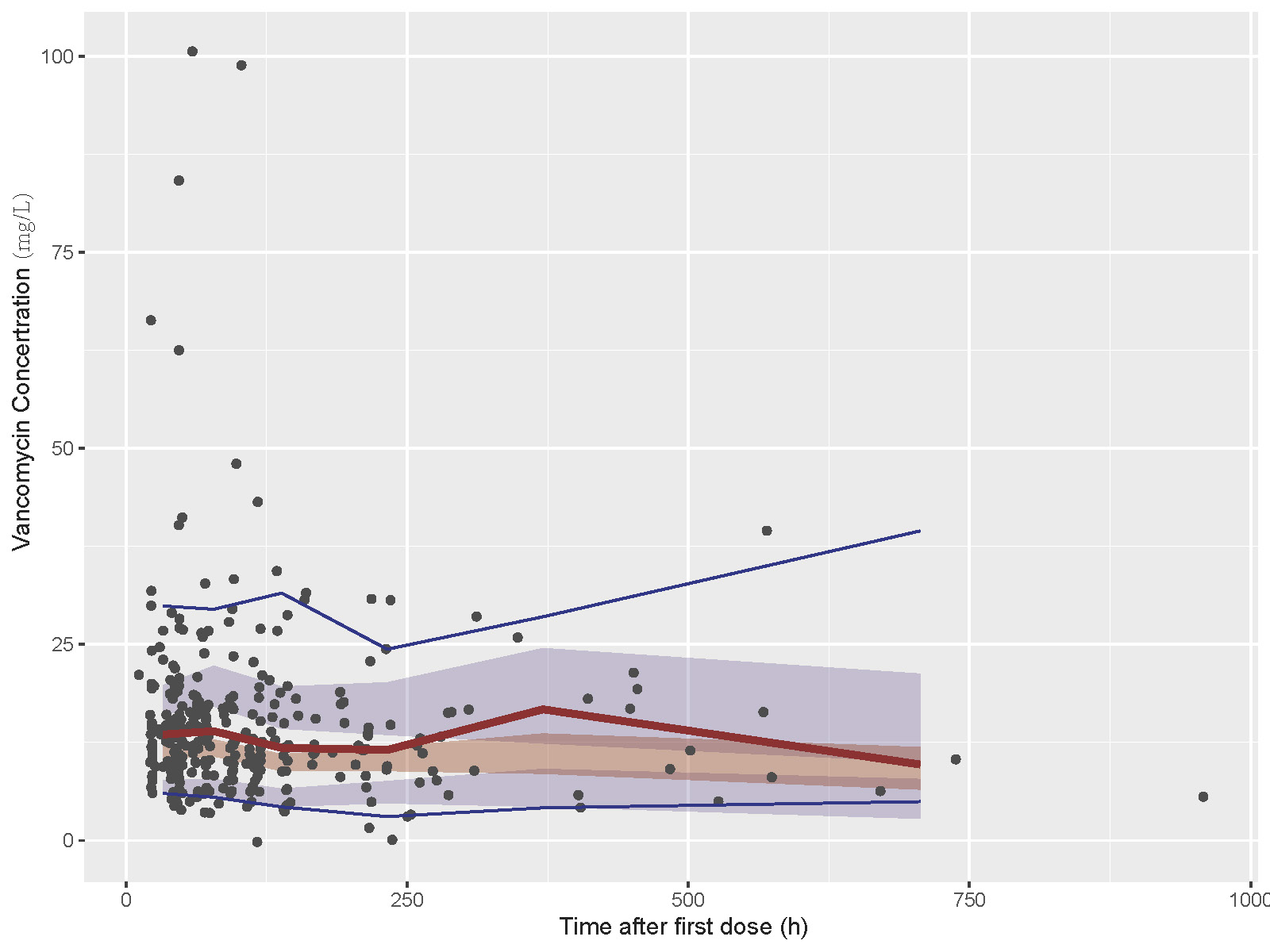

Supplement: Supplementary file 2 [file image2.jpeg]
