# Supplementary material for: External Evaluation of Vancomycin Population Pharmacokinetic Models at Two Clinical Centers
Source: Front Pharmacol. 2021 Mar 15;12:623907. doi: 10.3389/fphar.2021.623907 (PMC8058705; doi:10.3389/fphar.2021.623907)
Supplement: Supplementary file 3 [file DataSheet1.docx]

Table S1. Predictive performance of vancomycin models in external evaluation datasets

| Models | MDPE(%) | MAPE(%) | F_20_(%) | F_30_(%) |
| --- | --- | --- | --- | --- |
| Seay_et al.,1994 | 107.04 | 107.04 | 15.05 | 19.75 |
| Grimsley et al., 1999 | -51.91 | 55.75 | 13.48 | 22.57 |
| Capparelli et al., 2001 | -2.35 | 34.58 | 28.84 | 45.14 |
| Kimura et al., 2004 | -13.55 | 39.08 | 27.90 | 40.13 |
| Mulla et al.,2005 | -19.51 | 39.46 | 25.39 | 38.56 |
| Marqués Minñana et al., 2010 | 25.48 | 44.98 | 25.39 | 38.87 |
| Mehrotra et al., 2012 | 21.20 | 36.15 | 29.15 | 39.81 |
| Zhao et al., 2013 | -12.10 | 37.39 | 26.96 | 40.44 |
| Frymoyer et al., 2014 | -6.76 | 34.43 | 28.84 | 41.38 |
| Li et al ., 2017 | 4.84 | 30.52 | 35.42 | 49.53 |
| Sheng et al., 2017 | -37.40 | 58.07 | 15.36 | 24.45 |
| Song et al., 2017 | 0.12 | 42.21 | 21.94 | 35.11 |
| Chen et al., 2018 | -2.52 | 36.67 | 29.78 | 41.38 |
| Li et al., 2018 | 3.98 | 30.40 | 36.99 | 48.90 |
| Moffett et al., 2018 | 13.09 | 39.05 | 31.66 | 41.07 |
| Colin et al., 2019 | 8.27 | 34.87 | 28.84 | 43.57 |
| Germovsek et al., 2019 | 53.36 | 60.70 | 19.12 | 26.33 |
| Moffett et al., 2019 | -15.09 | 30.84 | 31.35 | 47.34 |

Table S2. Bayesian forecasting performance of different vancomycin PPK models

| Models | MDIPE  0 prior | MDIPE  1 prior | MDIPE  2 prior | MAIPE  0 prior | MAIPE  1 prior | MAIPE  2 prior | IF_20_  0 prior | IF_20_  1 prior | IF_20_  2 prior | IF_30_  0 prior | IF_30_  1 prior | IF_30_  2 prior |
| --- | --- | --- | --- | --- | --- | --- | --- | --- | --- | --- | --- | --- |
| Seay_et al.,1994 | 99.86 | 33.31 | 24.79 | 99.86 | 36.02 | 38.86 | 15.88 | 31.25 | 25.40 | 21.18 | 43.30 | 39.68 |
| Grimsley et al., 1999 | -56.83 | -38.09 | -25.60 | 60.92 | 43.72 | 37.55 | 9.41 | 18.75 | 27.78 | 18.24 | 32.14 | 43.06 |
| Capparelli et al., 2001 | -4.93 | 4.13 | 10.58 | 29.92 | 20.87 | 26.59 | 31.76 | 49.11 | 36.11 | 50.00 | 59.82 | 52.78 |
| Kimura et al., 2004 | -14.59 | -8.95 | -3.51 | 40.74 | 26.21 | 28.08 | 27.06 | 39.29 | 38.89 | 36.47 | 54.46 | 52.78 |
| Mulla et al.,2005 | -23.53 | -4.63 | -0.28 | 39.15 | 22.09 | 22.35 | 24.12 | 46.88 | 44.44 | 38.24 | 61.16 | 58.33 |
| Marqués Minñana  et al., 2010 | 23.79 | 31.72 | 62.60 | 43.97 | 48.01 | 62.60 | 25.29 | 23.66 | 19.44 | 35.88 | 34.82 | 26.39 |
| Mehrotra et al., 2012 | 23.30 | 15.13 | 17.16 | 36.26 | 26.50 | 31.41 | 29.41 | 38.39 | 40.28 | 37.65 | 52.68 | 48.61 |
| Zhao et al., 2013 | -17.11 | -0.40 | 4.14 | 35.19 | 15.55 | 28.22 | 28.24 | 56.25 | 38.89 | 43.53 | 65.18 | 51.39 |
| Frymoyer et al., 2014 | -9.06 | 2.72 | 10.42 | 34.15 | 16.12 | 25.57 | 30.00 | 54.46 | 37.50 | 44.12 | 66.96 | 58.33 |
| Li et al ., 2017 | -28.05 | -6.18 | 0.09 | 58.97 | 19.44 | 20.53 | 15.29 | 51.34 | 48.61 | 25.88 | 62.50 | 63.89 |
| Sheng et al., 2017 | 5.87 | 3.21 | 10.87 | 30.21 | 20.16 | 24.77 | 37.65 | 50.00 | 37.50 | 50.00 | 62.95 | 59.72 |
| Song et al., 2017 | 7.14 | 0.42 | 3.21 | 44.73 | 17.99 | 23.94 | 20.00 | 54.02 | 45.83 | 32.94 | 67.41 | 61.11 |
| Chen et al., 2018 | 4.80 | 2.90 | 9.29 | 29.12 | 15.76 | 22.24 | 38.24 | 57.14 | 47.22 | 51.76 | 68.75 | 59.72 |
| Li et al., 2018 | -5.29 | 3.64 | 11.53 | 36.17 | 14.32 | 26.44 | 32.35 | 57.14 | 38.89 | 42.94 | 66.96 | 56.94 |
| Moffett et al., 2018 | 15.98 | 4.22 | 16.28 | 42.02 | 16.33 | 26.24 | 31.76 | 54.02 | 37.50 | 38.82 | 66.07 | 56.94 |
| Colin et al., 2019 | 8.45 | 8.66 | 14.33 | 34.77 | 18.45 | 25.40 | 28.82 | 51.34 | 40.28 | 44.12 | 62.95 | 59.72 |
| Germovsek et al., 2019 | 52.57 | 30.82 | 30.59 | 60.07 | 35.10 | 34.91 | 15.88 | 33.93 | 36.11 | 22.94 | 45.98 | 44.44 |
| Moffett et al., 2019 | -15.16 | -2.64 | 6.00 | 30.72 | 14.23 | 22.03 | 28.82 | 62.05 | 43.06 | 47.06 | 73.21 | 58.33 |

Table S3. Predictive performance of vancomycin models with different covariates

| Models | MDIPE  0 prior | MDIPE  1 prior | MDIPE  2 prior | MAIPE  0 prior | MAIPE  1 prior | MAIPE  2 prior | IF_20_  0 prior | IF_20_  1 prior | IF_20_  2 prior | IF_30_  0 prior | IF_30_  1 prior | IF_30_  2 prior |
| --- | --- | --- | --- | --- | --- | --- | --- | --- | --- | --- | --- | --- |
| CMT1 | 10.33 | 5.96 | 22.44 | 63.81 | 23.68 | 30.11 | 14.12 | 45.54 | 31.94 | 19.41 | 57.14 | 50.00 |
| CMT1_MT | 8.49 | 4.11 | 10.65 | 34.64 | 21.49 | 27.71 | 27.65 | 48.66 | 40.28 | 44.71 | 59.38 | 51.39 |
| CMT1_WT1 | 4.57 | 5.78 | 18.70 | 33.16 | 22.06 | 28.64 | 34.71 | 45.54 | 34.72 | 45.29 | 58.48 | 52.78 |
| CMT1_WT2 | 5.96 | 5.52 | 19.71 | 42.23 | 23.27 | 30.01 | 22.94 | 43.75 | 33.33 | 34.12 | 58.48 | 50.00 |
| CMT1_SCR1 | 3.65 | 3.17 | 17.07 | 52.93 | 23.61 | 30.26 | 15.88 | 45.54 | 40.28 | 22.94 | 56.25 | 50.00 |
| CMT1_SCR2 | 6.12 | 8.36 | 21.55 | 74.15 | 27.92 | 36.49 | 10.59 | 44.20 | 34.72 | 17.65 | 52.68 | 43.06 |
| CMT2 | 3.49 | 4.82 | 16.85 | 63.96 | 24.81 | 28.41 | 14.12 | 41.96 | 34.72 | 19.41 | 55.80 | 52.78 |
| CMT2_MT | 4.29 | 2.44 | 8.14 | 35.06 | 22.56 | 28.80 | 30.00 | 45.98 | 38.89 | 43.53 | 58.48 | 50.00 |
| CMT2_WT1 | 2.94 | 4.78 | 17.93 | 32.80 | 23.04 | 28.85 | 34.12 | 43.30 | 33.33 | 43.53 | 57.14 | 52.78 |
| CMT2_WT2 | 4.45 | 4.75 | 18.28 | 40.86 | 24.22 | 29.25 | 22.94 | 42.86 | 33.33 | 33.53 | 57.14 | 51.39 |
| CMT2_SCR1 | 0.04 | 2.64 | 13.17 | 53.08 | 24.41 | 33.30 | 16.47 | 42.41 | 37.50 | 22.94 | 55.80 | 47.22 |
| CMT2_SCR2 | -7.95 | 3.68 | 13.49 | 73.68 | 29.22 | 34.75 | 11.76 | 38.39 | 34.72 | 17.65 | 50.45 | 48.61 |

CMT1, one compartment model; CMT2, two compartment model; MT, maturation model; WT1, allometric scaling model of WT; WT2, linear model of WT; SCR1, allometric scaling model of SCR; SCR2, linear model of SCR.
